# Evolving Network Analysis of S&P500 Components: COVID-19 Influence of Cross-Correlation Network Structure

**DOI:** 10.3390/e24010021

**Published:** 2021-12-23

**Authors:** Janusz Miśkiewicz, Dorota Bonarska-Kujawa

**Affiliations:** 1 Institute of Theoretical Physics, University of Wrocław, 50-137 Wroclaw, Poland; 2 Physics and Biophysics Department, Wrocław University of Environmental and Life Sciences, 50-375 Wroclaw, Poland; dorota.bonarska-kujawa@upwr.edu.pl

**Keywords:** network analysis, structural entropy, time series analysis, COVID-19

## Abstract

The economy is a system of complex interactions. The COVID-19 pandemic strongly influenced economies, particularly through introduced restrictions, which formed a completely new economic environment. The present work focuses on the changes induced by the COVID-19 epidemic on the correlation network structure. The analysis is performed on a representative set of USA companies—the S&P500 components. Four different network structures are constructed (strong, weak, typically, and significantly connected networks), and the rank entropy, cycle entropy, averaged clustering coefficient, and transitivity evolution are established and discussed. Based on the mentioned structural parameters, four different stages have been distinguished during the COVID-19-induced crisis. The proposed network properties and their applicability to a crisis-distinguishing problem are discussed. Moreover, the optimal time window problem is analysed.

## 1. Introduction

### 1.1. Literature Review

The crucial and most obvious features of economic systems are cooperation and interaction, e.g., supply chains, ownership dependencies, cooperation networks, and financial networks. The companies and their activities form complex networks of relationships and dependencies [1,2,3]. The idea that the economy can be considered as a complex system can be found at the end of the XX century in economic literature, e.g., [4]. The features of the network depend on various parameters, such as technology, law, regulations, culture, climate, weather, resources, and many other parameters. Among various aspects of economic activity, the financial market plays a special role. Besides fundraising for the business, they allow monitoring the condition of enterprises and even whole economic sectors. The stock market indices are regularly published and are considered to be not only a measure of the change of companies’ group values, but also a test of the economy’s status [5]. Therefore, within this study, the cross-correlations among stock time series of S&P 500 index components are analysed. This index was chosen because it is based on quotations of 500 shares of the largest stocks that trade on the New York Stock Exchange and NASDAQ. Considering the importance of those companies, as well as their variety and number, this set can be considered to be representative of the USA’s economy and, therefore, can be the basis of the analysis of cross-correlations among them. Particularly, these stocks can be analysed from the point of view of cross-correlation network structures formed by those companies. The cross-correlation analysis consists of two steps: the distance (usually based on linear correlation [6]) matrix calculation and its analysis by constructing a network of random matrix approaches [7,8,9]. The alternative cross-correlation analysis should be here mentioned. Recently, detrended cross-correlation analysis based on detrended fluctuation analysis (DFA) [10] and their modifications have been very popular; see the recent review [11], or the power law classification scheme [12]. However, the most popular strategy is based on the network construction and its structure analysis [13,14]. The most popular choice is the minimum spanning tree (MST) analysis [3,9,15,16,17,18,19,20,21]. MST application results primarily from the portfolio optimisation problem [22], but is also due to the proper recovering of the industrial sectors [3,6]. Besides MST analysis, the second most commonly used group of methods are those which construct networks based on assumed threshold [23]. This approach is used also in this paper—the distance matrix is filtered assuming that nodes are connected when the distance fulfills a given condition. Within this analysis, the properties of the most typical, weak, strong or significant correlations are investigated. The network generation procedures are described in detail in Section 3. The main aim of the paper is the description of the structural changes observed during the COVID-19 pandemic and their comparison to the changes in cross-correlation network properties during other, recently observed crises.

Another important aspect is the influence of the external parameters on the network structure. It is a truism to say that the state of the market depends on the macroeconomic situation. Particularly, the reaction of the market to crashes is also widely discussed from the point of view of network structure, e.g., [9,24,25], or in the analysis of globalization processes [26,27,28]. However, the present pandemic situation should not be considered as the typical shock but rather a change of the “economic environment”. The most important fact is that the pandemic was expected and induced serious changes in the economy. The government, due to the pandemic situation, introduced special rules lasting a relatively long time. The restrictions form special conditions which are expected to change the structure of cross-correlations among companies. The network structure of shares’ cross-correlations is the subject of interest of various studies [9,21,29]. A natural extension of time series analysis through the network methods is evolving network analysis, since the economy time series are non-stationary, and thus, should be described by an evolving network rather than a static network. Evolving network theory was initially applied to systems naturally described by network structures, such as social networks [30], scientific collaboration networks [31] and economy time series [8,25,32,33], to mention a few examples.

### 1.2. COVID-19 History

Although the COVID-19 epidemic is a contemporary event, for the convenience of future readers, a short description of the epidemic key points in the USA is presented below.

**December 2019** The first known cases have been identified in Wuhan, China.
**January 2020** The epidemic spreads to other provinces of China.
**February 2020** Italy is affected with a rapidly growing number of infected and fatal cases.
**March 2020** The USA overtakes China and Italy with the highest number of confirmed cases in the world.

The present situation is the subject of various studies. More detailed history and discussion on the influence of pandemic on stock markets from the standard time series analysis point of view can be found at [34,35,36,37,38].

### 1.3. Paper Structure

The present paper is organised as follows. Section 2 describes the data analysed. The Section 3 defines the methods used: the statistical distance, the network construction algorithms and the network parameters (node entropy, cycle entropy, averaged clustering coefficient and transitivity) calculated and analysed in the study. Section 4 presents the obtained results, including the evolution of the node and cycle entropy and the averaged clustering coefficient and transitivity. It is worth stressing that the parameters introduced here (the node and the cycle entropy) are sensitive to economic crises. Moreover, the performed analysis shows that the structure of the defined networks changes significantly in the crisis. The main outcome of the paper is the analysis of a representative group of USA companies and the observation of their reaction to changing economic situations. A very promising outcome of the study is that four periods during the COVID-19 pandemic are distinguished, which shows that the reactions to various factors are different and that this analysis is capable of observing this.

## 2. Data

The study is based on the S&P 500 index components’ daily time series in the interval from 2016.01.04 to 2021.03.26. There are a total of 1315 data points in each of the time series. The S&P500 index consists of the largest stocks of the New York Stock Exchange and NASDAQ (National Association of Securities Dealers Automated Quotations). Considering the importance of stock indices for the assessment of the state of the economy, we can conclude that the entities on which the S&P 500 index is based constitute a representative group that allows the observation of important processes taking place in the economy. Furthermore, this index is based on a broad range of companies of different sectors; therefore, it can be considered representative for the USA economy. The time interval is chosen such that it contains a period before the COVID-19 pandemic. It is worth noticing that the pandemic period is before the broad availability of vaccination, so the observed changes are the effects of the institutional response to the pandemic situation. The inclusion of different periods is particularly important because the algorithm of the study is new and has never been tested.

The time series were downloaded from the web page Available online: https://stooq.pl (accessed on 28 March 2021). Although the index is based on 500 quotes, after inspection of the data, 432 time series were chosen for the analysis due to missing data. The list of quotations used for the analysis is presented in Appendix A.

The time series were converted into logarithmic daily return time series (so-called log-returns) according to Equation (1),

(1) $$LogR{\left(A\right)}_{i}=\log\frac{a_{i}}{a_{i-1}},$$

where *A* represents the time series, and $a_{i}$ represents the i-th element of the time series *A*.

**Figure 1:**
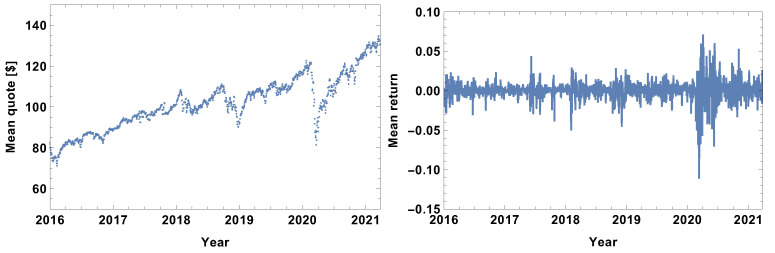
(**Left**) Evolution of the mean value of 432 quotes included in the study. (**Right**) Evolution of the averaged log-returns of 432 quotes included in the study.

The evolution of the mean value of stocks included in the study is presented in Figure 1. By analysing the evolution of the mean quote in Figure 1, it should be noticed that it has the form of a visible growing trend with periods of significant fluctuations. The fluctuations correspond to periods of rapid growth followed by a significant drop in value—price bubbles or crises resulting from external factors. The major fluctuations which should be pointed out are the beginning of 2016 (which is the result of two shocks, the Chinese stock market and USA stock market selloffs), the third quarter of 2017, the first quarter of 2018, the second half of 2018 (the cryptocurrency crash), minor fluctuations in the middle of 2019 and a dramatic drop at the beginning of 2020 related to the COVID-19 pandemic. In general, the fluctuations seen in the mean value of quotes are also present in the averaged log-returns evolution graph seen in Figure 1, particularly in the left and right plots, respectively. It is worth noting that the range of fluctuations at the beginning of 2020 was ≈0.2, while the others observed in the period 2016–2019 did not exceed 0.07; therefore, the fluctuations resulting from the pandemic shock were approximately 3 times bigger than the other shocks. Within the analysed period, COVID-19 is the dominating factor in financial markets.

## 3. Methods

Considering the fact that the evolution of the network structure is investigated in this study, the sliding window technique was applied. The essence of this method is that a fragment of a fixed length (the time window size) is selected from the time series. An analysis is performed for this fragment, and subsequently, the beginning and the end are shifted by one point and all calculations are repeated. The procedure is repeated until the time window reaches the end of the series.

The analysis carried out in this work can be divided into the following main steps:

1. Distance matrix calculations;
2. Network construction;
3. Network feature analysis.

### 3.1. Distance Matrix

The distance between the log-returns time series is calculated based on the ultrametric distance [26,27,39,40] as in Equation (2),

(2) $$DM{\left(A,B\right)}_{t,T}=\sqrt{\frac{1}{2}\left(1-\rho {\left(A,B\right)}_{t,T}\right)},$$

where the correlation $\rho {\left(A,B\right)}_{t,T}$ is calculated using Pearson correlation coefficient, as in Equation (3):

(3) $$\rho {\left(A,B\right)}_{t,T}=\frac{{\langle AB\rangle }_{t,T}-{\langle A\rangle }_{t,T}{\langle B\rangle }_{t,T}}{\sigma {\left(A\right)}_{t,T}\sigma {\left(B\right)}_{t,T}}.$$

where the indices ${\left(\right)}_{t,T}$ denote the interval (t,t+T). *T* stands for the time window size. The distance DM, when equal to zero, indicates a perfect linear correlation between time series, while a distance DM equal to one is obtained in the case of a lack of linear correlation (which does not mean that the time series are not correlated by other functions [39]).

In the literature, there is an alternative formulation of Equation (2), the ultrametric distance, which utilizes different normalization techniques [20]. Of course, the normalization does not affect the conclusions. The ultrametric distance DM is calculated for all possible pairs of time series, and the results are presented in the form of the distance matrix. The distance matrix DM is symmetrical due to the definition of the ultrametric distance Equation (2).

### 3.2. Network Construction

Considering the fact that each distance matrix contains $\frac{\left(n(n-1\right)}{2}$ different elements, here it gives 93096 different numbers. The analysis of the distance matrix requires the construction of higher-order structure—networks. Although in the literature the minimum spanning tree (MST) is one of the most popular structures [6,16,18,19,41,42], it imposes a very strong bias on the generated network. For example, due to the imposed tree structure, it is impossible to observe cliques, which are quite important elements of economic relationship analysis. In the case of MST analysis, with some additional effort, it is also possible to distinguish clusters [16], but such analysis is not straightforward due to the tree structure. MST analysis often distinguishes one prominent node, eg. [16,42], but in different network structures, the node could be a member of a clique and such a conclusion of its special role would be not possible.

Therefore, in this paper, the threshold method is used. The distances are categorised into defined groups, and, in each case, the network is constructed based on the appropriately filtered distance matrix.

Distance categorisation:

- Strongly connected time series—the companies are connected when the distance is shorter than the first quartile of the distances in the analysed distance matrix, so the network is built on a set of the 25% shortest links;
- Weakly connected time series—the companies are connected when the distance is longer than the third quartile of the distance in the analysed distance matrix, so the network is built on a set of the 25% longest links;
- The most typical connections—the companies are connected when the distance between them is longer than the first quartile and shorter than the third quartile of the distances in the analysed distance matrix, so the network is built on this set of 50% of the links;
- Significantly connected time series—the companies are connected in the network when the distance between them is shorter than the median of the distances in the analysed distance matrix, so the network is built on a set of 50% of the links.

The examples of the network generated in the study are presented in the Appendix B. Due to the huge number of graphs generated in the analysis (the time series length diminishes by the time window size) and the size of the networks, only a few examples are presented focusing on the state before the COVID-19 pandemic (July/August 2019) and two examples during the pandemic (March 2020 and August/September 2020).

On the other hand, the MST analysis allows the dominating node to be distinguished, usually with the highest number of links, eg. GE in [16]. However, this result partially depends on the imposed tree structure. In the threshold method, such situations are less probable, and a very high number of companies have a high number of links, so such prominent nodes are not observed.

### 3.3. Network Analysis

The last step of the analysis is the network parameter calculations. Considering the fact that, in the study, more than a thousand networks are constructed (due to the sliding window technique) and each network consists of 432 nodes, the direct analysis is tremendous. On the other hand, the general state of the system can be characterised by calculating appropriately chosen parameters.

The study aims to observe changes in the structure of the network of correlations. In the case of economic systems, some structures are of special interest. Usually one of the very first issues analysed is the leadership, or the presence of dominating companies, which are network hubs. The second most important structures are clusters that correspond to strongly cooperating companies or sets with strong mutual relationships, e.g., belonging to the same highly specialised sector, with the same ownership or sharing another common factor. The question of the presence of dominating companies is answered by the rank node analysis, which ranks nodes with respect to the number of links. It was shown in [22,32,42] that during crises, the dominating structure is a star-like network with a well-defined centre. On the other hand, in independently developing companies, one can expect that the statistical distances among time series would be similar (with some fluctuations). Moreover, the most interesting aspects, from the point of view of questions raised, are the changes in the network structure. Thus, the measure which properly exposes such structures and their changes is Shannon entropy. Therefore, the rank node distribution is characterised by information entropy; here it will be called **rank node entropy** and defined by Equation (4),

(4) $$SN=-\sum_{i\in L}p_{i}\ln p_{i}$$

where *L* represents the list of all observed ranks, and $p_{i}$ represents the probability of the i-th rank node.

The second feature investigated is the formation of particular structures, specifically triangles and cycles. The triangles expose the companies forming closely interacting groups; analogously, cycles are the groups with significant relationships (a chain of dependence). These two parameters are analysed by the calculation of transitivity and cycle entropy. The transitivity is defined as the fraction of all possible triangles in the graph.

(5) $$T=3\frac{\#triangles}{\#triads}$$

where triad indicates two edges with a shared vertex. **The cycle entropy** is defined as the information entropy of the cycle length distribution:

(6) $$SC=-\sum_{i\in C}p_{i}\ln p_{i}$$

where *C* indicates the list of all observed cycle lengths, and $p_{i}$ represents the probability of observing a cycle of the length *i*.

The last analysed network parameter is the clustering coefficient, which is the standard characteristic of the link density. Here, the averaged clustering coefficient is used, which is defined by Equation (7):

(7) $$C=\frac{1}{n}\sum_{v\in G}c_{v},\;\;\;c_{v}=\frac{2T(v)}{deg(v)(deg(v)-1)}$$

where T(v) is the number of triangles through node *v*, and deg(v) represents the degree of node *v*.

The last element of the analysis procedure to define is the time window length. Considering the analysis of daily time series, three time window lengths have been chosen: 5 days, 20 days, and 60 days, which correspond to the week, month, and quarter periods, respectively.

A summary of the analysis algorithm is as follows:

1. Choose the representative set of companies (shares);
2. Verify the integrity of the time series and their length (should be identical);
3. Normalise the time series by converting them to the daily log-return time series;
4. Choose the time window size;
5. For each of the time series, starting at the beginning, take the interval of the time window length and calculate the time series correlation (distance) matrix;
6. Based on the correlation matrix, generate the network. Here, four possible strategies are considered: (i) strongly, (ii) weakly, (iii) most typical, (iv) significantly connected networks, so the following steps should be repeated for each network type;
7. Calculate the network’s characteristics: rank entropy, cycle entropy, averaged clustering coefficient and transitivity;
8. Move the starting point by one point and repeat steps 5–8. Continue until the end of the time series length is reached.

Finally, the time evolution of the network characteristics is received and discussed.

## 4. Results

### 4.1. Week Size Time Window, T=5d

**Figure 2:**
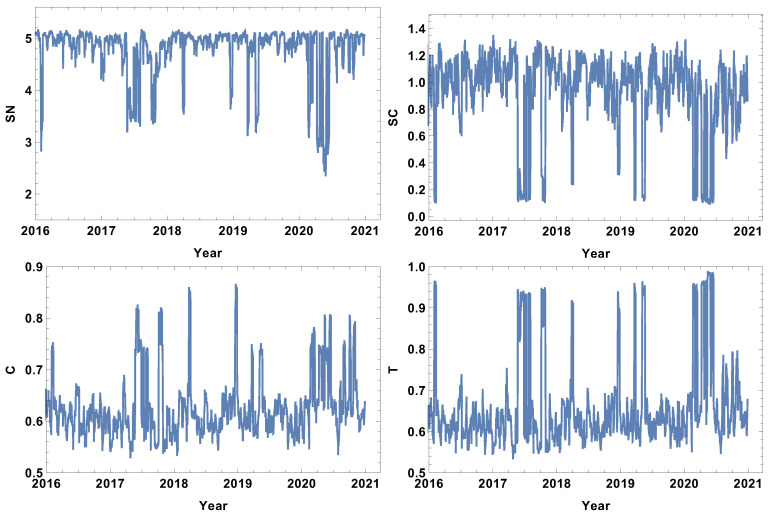
Network feature evolution in the case of the strongly connected companies, i.e., the distance between them is shorter than the first quartile of distances in the analysed distance matrix. The time window T=5d. The top left figure represents rank entropy, the top right represents cycle entropy, the bottom left represents the averaged clustering coefficient, and the bottom right represents transitivity.

**Figure 3:**
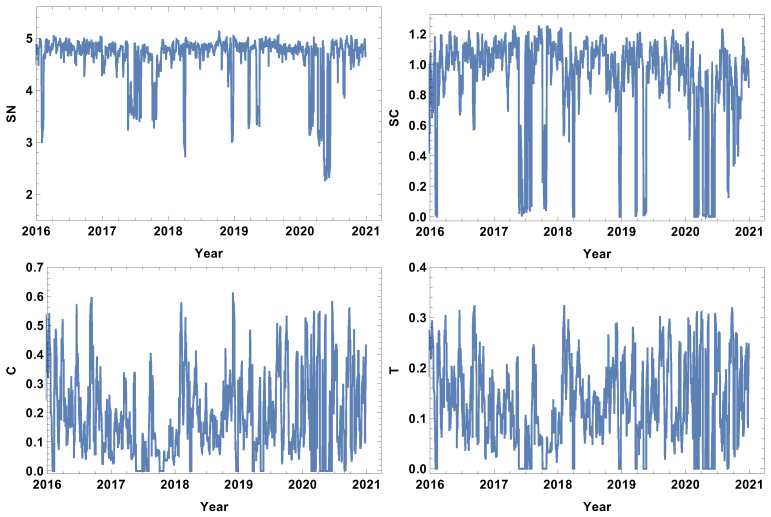
Network feature evolution in the case of the weakly connected companies, i.e., the distance between them is longer than the third quartile of the distances in the analysed distance matrix. The time window T=5d. The top left figure represents rank entropy, the top right represents cycle entropy, the bottom left represents the averaged clustering coefficient, and the bottom right represents transitivity.

**Figure 4:**
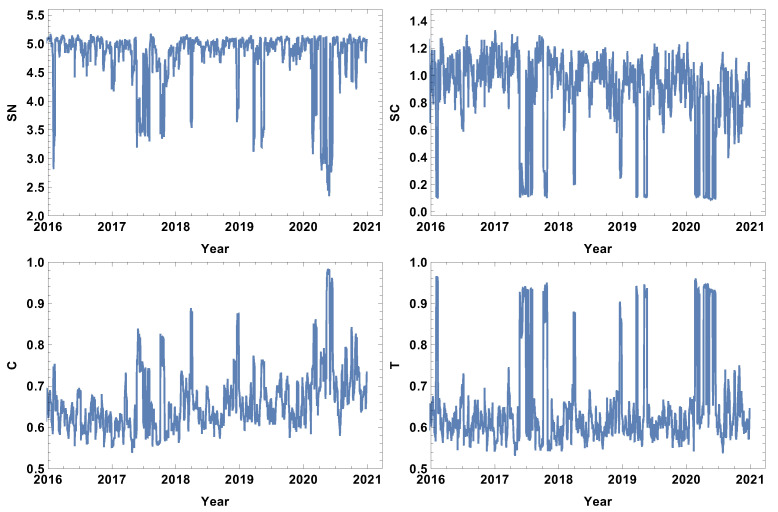
Network feature evolution in the case of the most typically connected companies, i.e., the distance between them is within the interval between the first and the third quartile of the distances in the analysed distance matrix. The time window T=5d. The top left figure represents the rank entropy, the top right represents cycle entropy, the bottom left represents the averaged clustering coefficient, and the bottom right represents transitivity.

**Figure 5:**
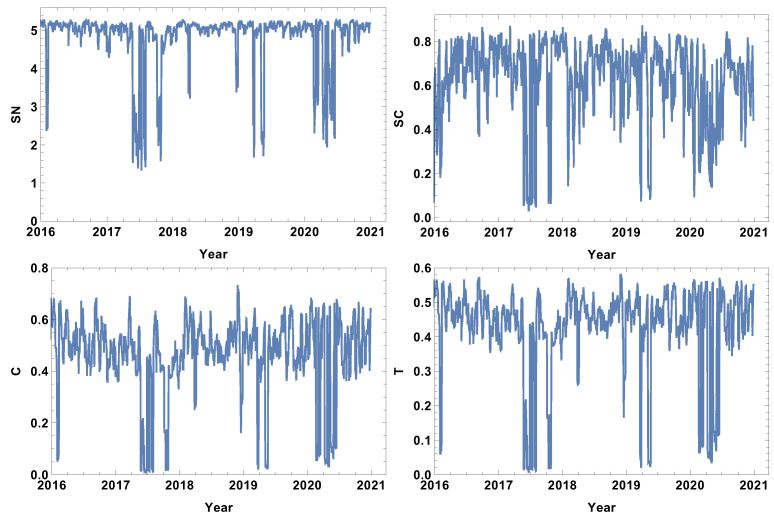
Network feature evolution in the case of the significantly connected companies, i.e., the distance between them is shorter than the median of the distances in the analysed distance matrix. The time window T=5d. The top left figure represents rank entropy, the top right represents cycle entropy, the bottom left represents the averaged clustering coefficient, and the bottom right represents transitivity.

The analysis begins with the shortest time window T=5d. The evolutions of the strongly, weakly, most typically, and significantly connected network properties are presented in Figure 2, Figure 3, Figure 4 and Figure 5. As was mentioned in Section 3.2, each of the structures is focused on different features of the system. The first network presented, which is of strongly connected companies, is built under the assumption that the companies are connected when the distance between them is shorter than the first quartile of the distances in the given distance matrix. The evolution of rank entropy, cycle entropy, averaged clustering coefficient, and the transitivity in the considered period are presented in Figure 2.

In the rank entropy evolution chart, one can distinguish the maximum state, which corresponds to the periods of “normal” trading, i.e., beyond crisis periods. Furthermore, similar observations can be made for the other rank entropy graphs independent of the time window size and the network structure considered; in all of them, stable maximum entropy is observed, suggesting that there exists a stable level of the rank distribution entropy. Besides the presence of the maximum rank entropy state, there are periods when the rank entropy is clearly smaller. At the beginning of 2016, which corresponds to the first fluctuation period in the considered interval, the rank entropy decreases from the value of 5 to 3. An analogous change is observed in the middle of 2017, the first quarter of 2018, and the crisis moment of 2019. The lowest values of rank node entropy are observed in the second quarter of 2020, which correlates with the development of the COVID-19 pandemic. Moreover, the evolution of the rank entropy reflects different stages of the reaction to the pandemic. At the end of 2019, it was obvious that the pandemic would spread all over the world, so in the beginning of January 2020 the first decrease in the rank entropy is observed as the result of news. Afterwords, the network structure began returning to the typical state. However, when the first cases were observed in USA, and consequently, the number of hospitalised persons began rapidly growing, the rank entropy decreased, reaching the lowest observed value and indicating significant changes in the network structure of the strongly connected companies.

The cycle entropy is focused on the cycle distribution length. In contrast to the rank entropy, the week time window analysis of the cycle entropy (Figure 3) does not show a clear stable maximum state. The cycle entropy in the period between crushes takes a value in the interval between 0.7 and 1.3, but during crises, the cycle entropy decreases to the value 0.1 (high fluctuation periods). It seems that during crises, most of the cycles are broken and the cycle entropy takes a very low value. In contrast to the rank entropy, the cycle entropy does not allow the severity of crises to be measured, since the same level is obtained for the crises at the beginning of 2016, the middle of 2017, the first and second quarter of 2019 and the COVID-19-induced crisis in 2020. Therefore, the cycle entropy achieves the lowest observed values relatively faster.

Besides the new measures introduced here (rank entropy and cycle entropy), the standard network parameters—averaged clustering coefficient and transitivity—are also sensitive to the COVID-19-induced crisis. Particularly, the transitivity obtains a very high value at the beginning of 2020. However, similarly to the cycle entropy, the transitivity does not allow the severity of the crises to be measured. The COVID-19 crash is characterised by similar values as the other crashes. The averaged clustering coefficient seems to be a less useful parameter in measuring crises strength because the highest values are observed in the first quarters of 2018 and 2019. When analysing the results of the network of the strongly correlated companies, it should be taken into account that this network is based on 25% of the most correlated time series, so this assumption may induce a dichotomous state of the network structure: crises and not crises.

The evolution of the weakly connected companies’ network parameters seems to be complementary to the strongly connected time series. In this case, the analysis is focused on the structure of the networks connected by a long statistical distance. The results for the week time window are presented in Figure 3. Analogously to the case of strongly connected companies, the rank entropy graph has a clear maximum value (≈5). The crash periods are correlated with a significant decrease in the rank entropy, and, similarly to the already discussed case, the lowest rank entropy is observed in the first and second quarter of 2020, which corresponds with pandemic development in the USA. The decrease in the rank entropy indicates the increase in differences in rank distributions, which is a quite natural process—during crises, a star-like network is dominating [22,32,41,42]. The two other coefficients analysed, i.e., the averaged clustering coefficient and transitivity, present a very noisy graph. In this very short time window, the fluctuations are dominating and do not allow any particular network features to be distinguished.

The results of the most typically connected companies are presented in Figure 4. In this case, the analysis is focused on the companies among which the distance is within the first and third quartile. Therefore, the network excludes extreme cases but shows the structure of typical connections among companies. The rank entropy graph, similar to the already discussed cases, is sensitive to significant price fluctuations. During crashes, the rank entropy value visibly decreases. On the other hand, the network during normal trading is characterized by rank entropy SN≈5. In contrast to the strongly and weakly correlated networks, after the large decrease in rank entropy related to the COVID-19 pandemic, the system does not return to the standard state at the level SN≈5, but is in the interval SN∈(4,4.5). Of course, the initial shock was the strongest one, particularly as it was followed by strong restrictions. However, in the second half of 2020, the situation did not return to the normal situation as the economy was still affected by the pandemic, and the rank entropy of the typically connected company network seems to be sensitive to this fact. The cycle entropy for the typically correlated company network, similar to the strongly and weakly correlated company networks, attains its minimal value at the time of large fluctuation periods SC≈0.1, indicating that crashes very strongly affect the cycle length structure. Averaged clustering coefficient evolution, in contrast to the weakly collected time series network, is sensitive to crises, having local maxima at the stock market crises. The essential feature of this result is that the highest local maximum is correlated with the COVID-19 period, when the largest fluctuation appeared. The latter observation is important because it shows that this network structure is sensitive not only to the presence of fluctuations but also to its magnitude. In the case of the last parameter, transitivity, the graph evolution seen in Figure 4 shows that, during crises, the structure of the network changes significantly (clearly distinguished local maxima), achieving transitivity twice as big compared to the standard fluctuation level.

The evolution of the network parameters of significantly connected companies is presented in Figure 5. In this case, the analysis concentrates on highly correlated companies, including those which are the most correlated. This is also the network based on half of the correlations. The features of the significantly connected network are slightly surprising, since the local minima related to the COVID-19 pandemic period are not the deepest minima. Beginning the analysis of this type of network with the rank entropy graph, it is seen that the smallest values of SN are observed in the 2017 crisis. The difference between the local minima in 2017 and 2020 is ≈0.6, which is not very high when comparing it to the maximum level SN≈5. A similar observation is made on the cycle entropy graph, in which SC fluctuates significantly even beyond the periods of crises. This indicates that the time window length seems to be too short to smooth the system fluctuations. The averaged clustering coefficient graph of significantly connected companies at the periods of crises achieves a value close to zero, which means that during a crisis, the cliques are almost whipped out of the network. The transitivity of the significantly connected time series supports the observations made on the averaged clustering coefficient graph, while the minima correspond to crisis periods. However, the changes in the network structure are so significant that the transitivity nearly reaches zero, indicating that triangles of correlated companies are very rare during crises.

To summarise the week time window analysis, the COVID-19 effect is observed at the beginning of 2020, as seen in the decrease in the rank entropy value. It is worth noting that the reaction of the rank entropy evolution to the price fluctuation does not depend on the type of network considered. Of course, the obtained results differ in details, but for all considered cases, the rank entropy graph has a “maximum level” describing normal stock exchange market activity and significantly decreases with large fluctuations, indicating changes in the rank node distribution.

### 4.2. Month Size Time Window, T=20d

**Figure 6:**
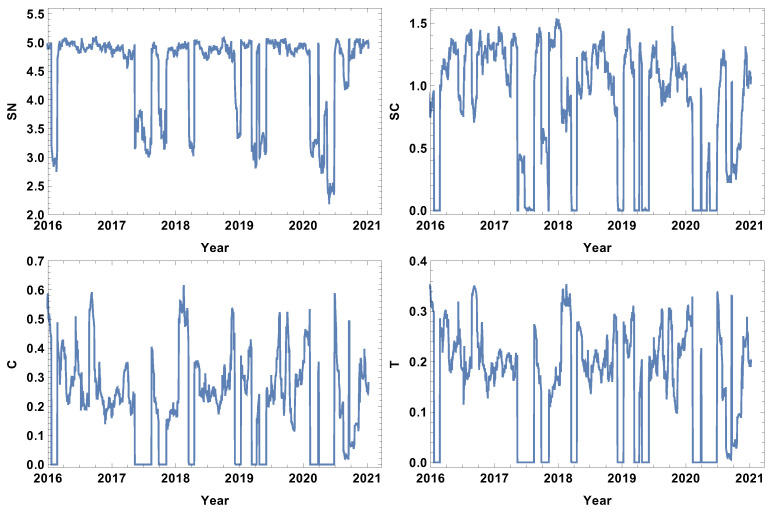
Network feature evolution in the case of the strongly connected companies, i.e., the distance between them is shorter than first quartile of distances in the analysed distance matrix. The time window T=20d. The top left figure represents rank entropy, the top right represents cycle entropy, the bottom left represents the averaged clustering coefficient, and the bottom right represents transitivity.

**Figure 7:**
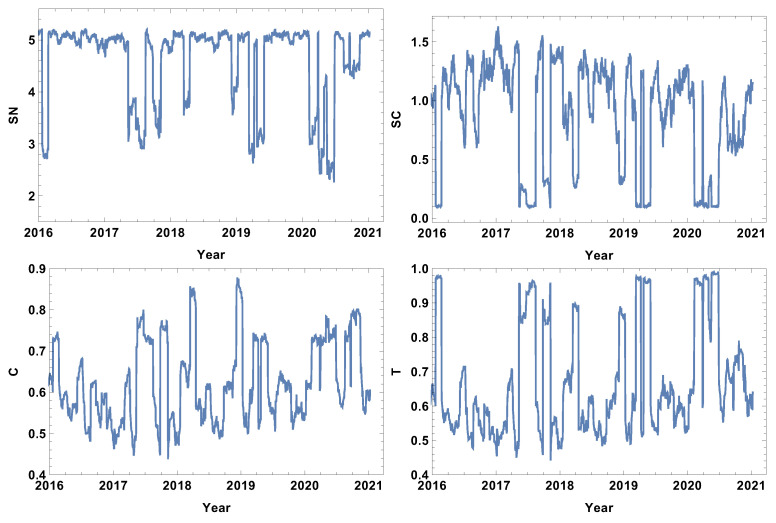
Network feature evolution in the case of the weakly connected companies, i.e., the distance between them is longer than the third quartile of the distances in the analysed distance matrix. The time window T=20d. The top left figure represents rank entropy, the top right represents cycle entropy, the bottom left represents the averaged clustering coefficient, and the bottom right represents transitivity.

**Figure 8:**
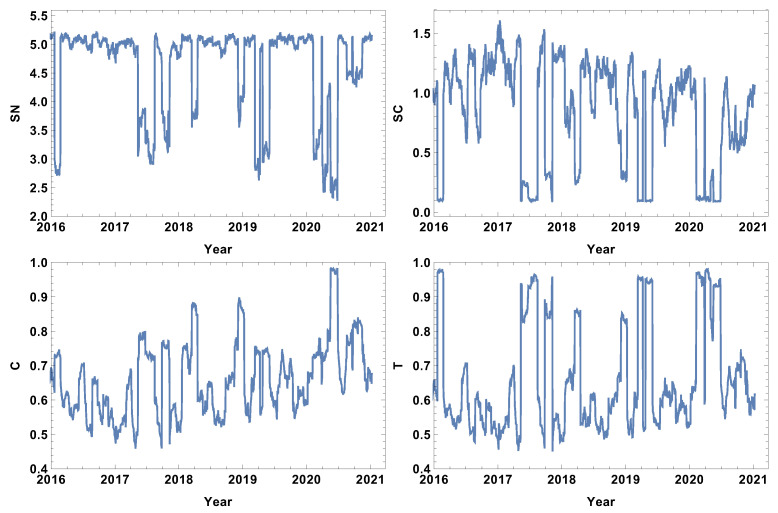
Network feature evolution in the case of the most typically connected companies, i.e., the distance between them is within the interval between the first and the third quartile of the distances in the analysed distance matrix. The time window T=20d. The top left figure represents rank entropy, the top right represents cycle entropy, the bottom left represents the averaged clustering coefficient, and the bottom right represents transitivity.

**Figure 9:**
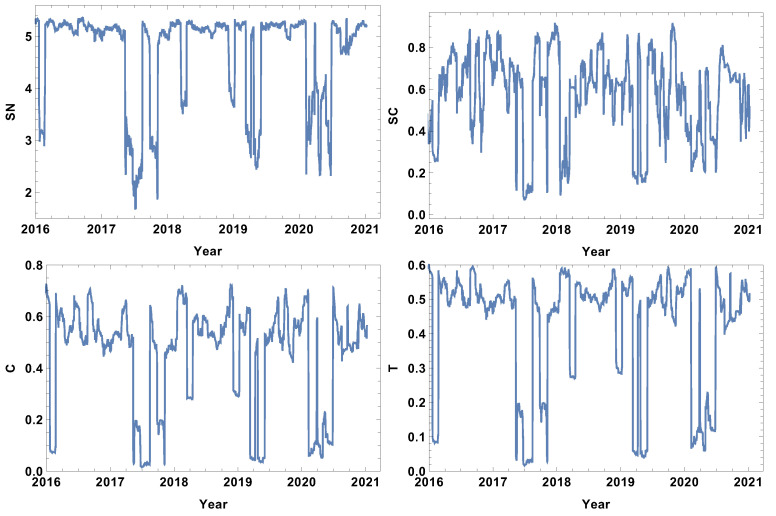
Network feature evolution in the case of the significantly connected companies, i.e., the companies are connected when the distance between them is shorter than the median of the distances in the analysed distance matrix. The time window T=20d. The top left figure represents rank entropy, the top right represents cycle entropy, the bottom left represents the averaged clustering coefficient, and the bottom right represents transitivity.

The next considered time window size was the month size time window (T=20d). The results are presented in Figure 6, Figure 7, Figure 8 and Figure 9. The first and most visible observation of the month window size analysis is the reduction of local fluctuations compared to the results obtained for the week time window size, as seen in Figure 3, Figure 4 and Figure 5.

The network features of the strongly correlated companies are presented in Figure 6. The most obvious observation is the decrease in the noise level compared to the week time window size analysis. The rank node entropy, as in the previous case, has a clear maximum level (SN≈5), which corresponds to the period of trading without significant price fluctuations. However, the intervals of decrease are not in the form of rapid and large oscillations, but have a shape of intervals, indicating that the change of structure was observed in the whole crisis period. The significant decrease in the rank entropy by 2.5 or more indicates a rapid and serious change of network structure. At the crash, the network reconstructs immediately to a new state characterised by much lower rank entropy. It can be seen that, for the month resolution analysis of the group of strongly correlated companies, the COVID-19-induced crises had three stages in which the rank entropy decreased abruptly. The three other network parameters, i.e., cycle entropy, averaged clustering coefficient, and transitivity, also decrease at this crisis; however, the value does not depend on the crisis severity, but they achieve the lowest possible value equal to zero indicating that during a crisis the higher-order structures, such as loops, triangles or clusters, do not exist in the network of the most-correlated companies.

The weakly connected time series network features are presented in Figure 7. Similarly to the strongly correlated network case, the rank entropy evolution for the network of the weakly correlated companies allows the crises intervals to be distinguished. The lowest rank entropy is observed during the COVID-19 crisis. The cycle entropy graph in the case of weakly connected companies also has a visible drop of cycle entropy value, but the lowest observed value is SC≈0.1, which indicates that only a few types of cycles are present in the network. In view of the already discussed cases, the averaged clustering coefficient evolution is very interesting. The weakly correlated companies’ network structure follows a different pattern than the already discussed network structures; during crises, the averaged clustering coefficient takes a very high value, indicating the strong clustering of companies. The same observation can be made by the analysis of the transitivity evolution graph; the maximum value is obtained at the crises periods, so weakly correlated companies form a high number of triangles. This finding correlates with the results of the network of the strongly correlated companies, where, during crises, the complex structures disappear; they emerge in the weakly correlated network.

The results of the analysis of the network of typically connected companies are presented in Figure 8. The rank entropy graph for the network of typically connected companies is similar to the already discussed cases, which properly indicates the crisis periods when the rank entropy decreases significantly. During the COVID-19-induced crisis, three stages of rank entropy value can be distinguished. However, they are not so separated as in the case of the strongly connected company network Figure 6. The cycle entropy graph supports the previous findings: during crises, cycles almost disappear from the network. On the other hand, the clustering coefficient is rather high in the crisis period. The highest averaged clustering coefficient is observed during the second quarter of 2020 (*C* is close to 1). An analogous observation can be made on the transitivity when the number of triangles is very high during crises. An interesting observation is made while comparing the averaged clustering graph with the transitivity plot. In the first half of 2020, the transitivity achieved a high value (the first and second quarter of 2020), while the averaged clustering coefficient takes a value close to one during the second quarter of 2020, at the time when the pandemic became very serious and stronger restrictions were imposed. This observation is meaningful since the transitivity is very sensitive to price fluctuations and immediately goes to a value close to one, while the clustering coefficient is more robust, allowing us to not only observe the fact that the network structure has changed but also relate the changes to the crisis severity.

The network results of the significantly connected companies are presented in Figure 9. Here, the companies are connected in the network when the distance between them is smaller than the median of the distance matrix, so the investigated group consists of relatively strongly correlated companies, and the analysis is based on half of the possible links. Comparing the results obtained in T=20d and in T=5d, it can be observed that the fluctuation level is significantly reduced, but the main findings are also valid for this analysis. The first important observation is that, in the case of the network of significantly connected companies, the smallest value of rank entropy occurs in the middle of 2017. This means that, from the point of view of the strongly connected companies, the unexpected market fluctuations are much worse than even severe but predicted; the rank entropy decrease during the COVID-19 crisis is not so strong. The cycle entropy graph is still difficult to interpret because it is hard to indicate a clear relationship between cycle entropy value evolution and the crash history. It is probable that the time window is too short and the fluctuations of the system are still hiding the crisis influence. At the graph of the average clustering coefficient evolution, two states can be distinguished—the normal trading period when C∈(0.45,0,75), and the states of significantly lower value C∈(0,0,4), which corresponds to the crash interval. The market fluctuations observed at the beginning of 2016, the middle of 2017, the first two quarters of 2019, and COVID-19 had similar effects on the averaged clustering coefficient, which became nearly zero at those periods. The main features observed in the averaged clustering graph Figure 9 are also present in the transitivity graph in Figure 9, with the difference that the numerical values characterising normal trading periods and crashes are slightly different. The transitivity of the network of significantly connected companies for intervals without spectacular events are in the range T∈(0.4,0.6), while during crashes, these values decrease as far as zero. Similarly to the rank entropy and the averaged clustering coefficient Figure 9, the transitivity in 2017 went even lower than during the COVID-19 crisis, indicating slightly weaker changes despite much more significant price fluctuations. Once again this raises the question about the importance of the shock expectations. The correlated companies could react similarly, so the network structure is not completely changed.

### 4.3. Quarter Size Time Window, T=60d

**Figure 10:**
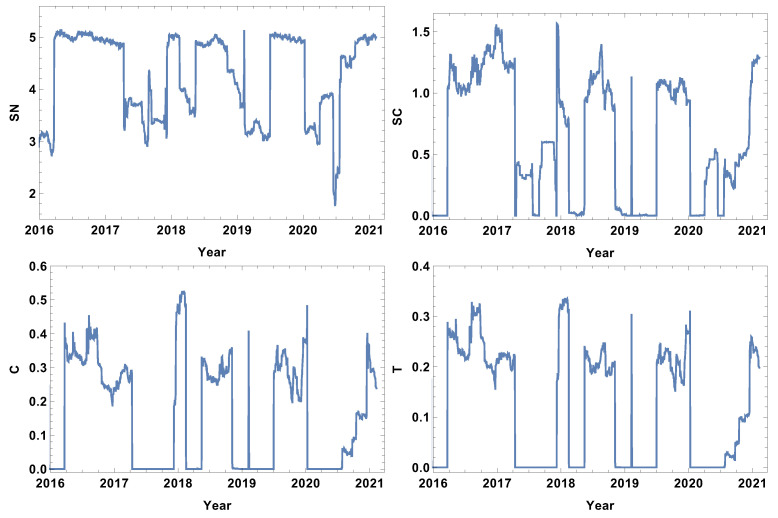
Network feature evolution in the case of the strongly connected companies, i.e., the distance between them is shorter than first quartile of the distances in the analysed distance matrix. The time window T=60d. The top left figure represents rank entropy, the top right represents cycle entropy, the bottom left represents the averaged clustering coefficient, and the bottom right represents transitivity.

**Figure 11:**
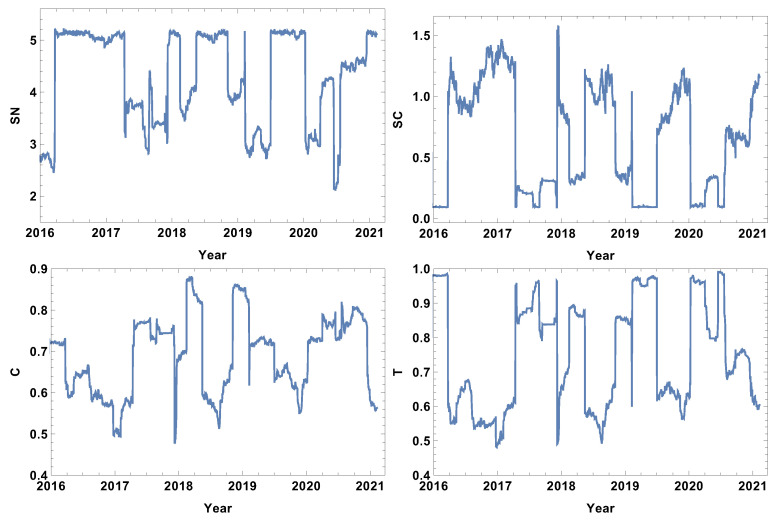
Network feature evolution in the case of the weakly connected companies, i.e., the distance between them is longer than the third quartile of the distances in the analysed distance matrix. The time window T=60d. The top left figure represents rank entropy, the top right represents cycle entropy, the bottom left represents the averaged clustering coefficient, and the bottom right represents transitivity.

**Figure 12:**
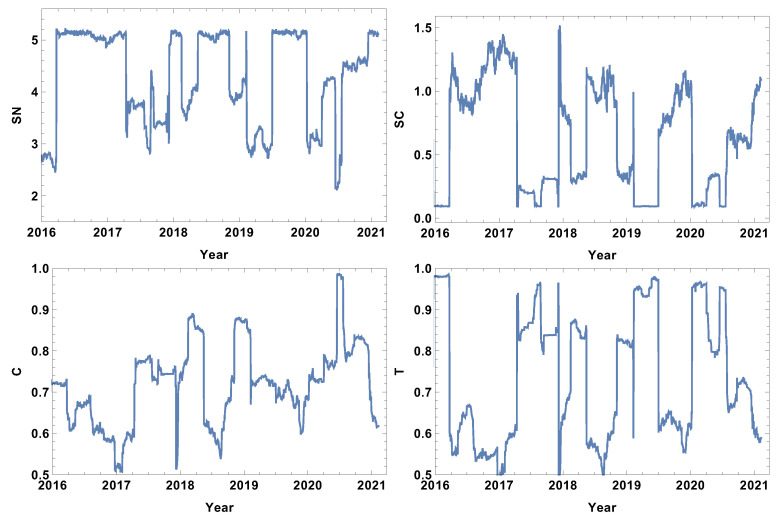
Network feature evolution in the case of the most typically connected companies, i.e., the distance between them is within the interval between the first and the third quartile of the distances in the analysed distance matrix. The time window T=60d. The top left figure represents rank entropy, the top right represents cycle entropy, the bottom left represents the averaged clustering coefficient, and the bottom right represents transitivity.

**Figure 13:**
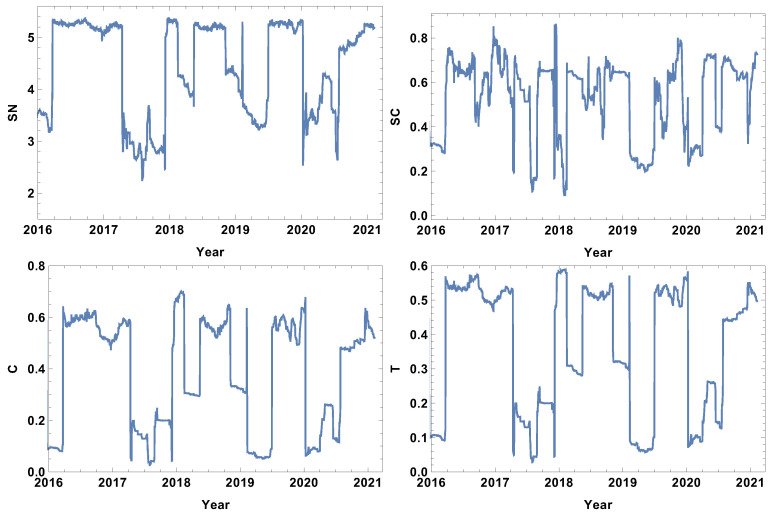
Network feature evolution in the case of the significantly connected companies, i.e., the distance is shorter than the median of the distances in the analysed distance matrix. The time window T=60d. SN≈0.9.

The results of the analysis for the quarter time window size T=60d are presented in Figure 10, Figure 11, Figure 12 and Figure 13. The quarter time window size is the longest time window considered in this study. As one can expect, the extension of the time window size filters the higher frequency fluctuations, allowing only the long-lasting correlations to remain, since each of the points in the graph is based on the cross-correlation distance calculated by the interval of 60 consecutive log-returns.

The network features of the strongly correlated companies are presented in Figure 10. The extension of the time window size resulted in clarifying the main features of the rank entropy graph, including the presence of a base level which describes the normal trading periods when the rank entropy is SN≈5. The crash periods demonstrate a significantly lower value of rank entropy of SN≈3. Considering the aim of the study, the most interesting evolution is the evolution of network parameters in 2020. The rank entropy plot shows that the network structure switched between three stages. The first stage was observed in the first quarter of 2020, when the pandemic was expected in the USA, and, due to the situation in China, the supply chain was affected. In the second quarter of 2020, the rank entropy increased to SN≈4, so the fact that the pandemic was expected resulted in some increase of the rank structure complexity when it came to the USA. However, as the situation developed and became severe in the first half of 2020, the rank entropy dropped up to SN≈ 2–2.4. The interesting finding is that the system adapted to the present situation, and at the end of 2020, the rank entropy returned to the level of the normal trading time SN≈5. The cycle entropy graph, in contrast to the rank entropy plot, does not have a stable value for normal trading, but during high fluctuation periods, the cycle entropy drops as far as zero. Very similar observations can be made on the averaged clustering coefficient and transitivity, suggesting that in the group of the most correlated companies, the higher-order structures are not present during crises. This is a new observation not previously discussed in the literature. By further analysing the cycle entropy graph, Figure 10, during the COVID-19 pandemic, it can be observed that after the initial drop of the cycle entropy in the first quarter of 2020, in the second quarter, the cycle entropy increases up to a value of SC≈0.5, which corresponds to the temporary increase of the rank entropy in the same period. This supports the expressed idea that, despite objective difficulties, the system is trying to adapt to the new situations. After the short decrease in the middle of 2020, the cycle entropy increases, reaching a value at the beginning of 2021 of SN≈1.3. The averaged clustering plot and transitivity graph follow a similar sequence, differing only in minor details during the periods beyond crises, but during the crises, values of both parameters decrease up to zero indicating disappearing complex structures among the most correlated companies. It should be stressed that the latter finding is observed in a very long time window, which means that during crises, long correlations do not form a complex structure.

The evolution of the network parameters of the weakly correlated companies is presented in Figure 11. The rank entropy evolution can be clearly divided into two categories: the crisis periods and the time beyond. During crises, the rank entropy decreases. The lowest value is observed at the beginning of 2016, when SN≈2.5, and in the middle of 2020, when SN≈2. Similar to the previous case discussed, the COVID-19 crash period can be divided into three stages: the change of SN from 5 to 3, then, in the second quarter, the rise to 4.1 and the significant drop in the middle of 2020 to 2. Afterwards, the rank entropy returns to its typical value. This observation supports the hypothesis that the network structure responded to the information of the incoming pandemic, then adopted to the situation and strongly reacted to the quick increase of affected cases and restrictions. The cycle entropy graph also supports the hypothesis of three COVID-19 stages. However, in SC, the first and the second stage is characterised by a very low value of cycle entropy SC≈0.1, with a short rise in SC during the second quarter of 2020 to the value SC≈0.3. The averaged clustering coefficient and transitivity show that, during crises, for the network of the weakly connected companies, the dominating structures are densely connected groups, since both parameters reach values close to the maximum, particularly for the transitivity where T≈1 is observed for all crashes in the analysed intervals. The long time window reduces the fluctuations present in shorter time windows (T=5d,T=20d) such that it is possible to analyse the evolution of the weakly correlated network structure.

The most typically connected time series network features are presented in Figure 12. The rank entropy graph shows that the most typically connected companies beyond the crises periods form networks, the rank entropy of which is SN≈5. During crises, the rank entropy decreases significantly, e.g., in the crisis of 2016 SN≈2.4, and during the COVID-19 crisis, SN≈2. In the evolution of SN in 2020, four stages can be distinguished. The first stage was in the first quarter of 2020, where SN≈3 as the stock market was scared by news from China. The second was in the second quarter of 2020, where SN≈4.2 when COVID-19 entered the USA. The third was observed in the middle of 2020, when SN≈2 when the situation worsened significantly and serious restrictions were imposed. The fourth stage lasted through the second half of 2020 when SN≈4.6. The cycle entropy graph in Figure 12 differs from the rank entropy in that, during crisis, cycle entropy is likely to reach a very low value of SC≈0.1. However, similar to the rank entropy, the four stages of the COVID-19 crisis can be distinguished by the difference that, during the first quarter in 2020 and in the third stage in half of 2020, the cycle entropy went to the same value SC≈0.1. Another important feature of the cycle entropy graph is that, in contrast to the rank entropy plot, the cycle entropy has a nontrivial evolution between crashes, showing the increasing complexity of the typically connected network. The two other parameters (averaged cluster coefficient and transitivity) show that, during crises, the most typically connected companies form a cluster or a structure close to it. Particularly high values of the averaged clustering coefficient of SN≈0.9 were observed in the middle of 2020 when the pandemic situation was significantly worsened. The transitivity graph in Figure 12 supports the observation of four stages in the COVID-19 crisis in 2020 made in the analysis of the rank entropy and cycle entropy of the most typically connected companies.

The analysis of the significantly connected companies over the quarter time window size of T=60d are presented in Figure 13. The rank entropy evolution for the network of significantly connected companies can be divided into two stages: the crisis period, when the rank entropy takes low values of SN∈(2,4.6), and the intervals beyond crises, when SN is relatively stable, such as SN∈(5,5.4). Although in 2020 the decrease resulting from the COVID-19 crisis is clearly visible, the stages observed in the cases of the networks of the strongly, weakly, and typically connected companies cannot be distinguished. The cycle entropy plot in Figure 13 shows that the cycle length distribution entropy is rather difficult to interpret in view of crisis presence and its severity. The opposite observation can be made for the averaged clustering coefficient and transitivity graphs. In these two graphs, Figure 13, the crises periods are characterised by a clear decrease of those parameters. During the COVID-19 crisis, four stages can be distinguished, similar to the networks of the strongly, weakly, and typically connected companies. The 2020 crisis began with a decrease in the averaged clustering coefficient from C≈0.65 to C≈0.04, and this coefficient remained at this value through the first quartile of 2020. Afterwards, it increased to 0.24 and kept this value until the middle of 2020, when it decreased to 0.1. Then, in the middle of the third quarter, it increased to the value C≈0.45. Analogous evolution is observed in the transitivity graph Figure 13 with slightly different values but with identical periods.

## 5. Conclusions

The presented study analysed the impact of the pandemic on the structure of cross-correlation networks among the most important companies on S&P500 components. The stock market crashes strongly influence cross-correlation network structure. Four different networks have been introduced and investigated: strongly, weakly, most typically, and significantly connected companies. The first 2 networks are based on 25% of links while the 2 latter networks are constructed on 50% of links. In general, all constructed networks are sensitive to large price fluctuations. Of particular interest was the crisis induced by the COVID-19 pandemic, where four stages of the market reaction were distinguished. It is worth stressing that the observed changes in the network structure can be related to particular features of the 2020 situation. The essential result is that the discussed changes can be quantified by rank entropy and cycle entropy measures, as well as the standard network parameters, such as the averaged clustering coefficient and the transitivity. The networks of strongly connected companies react in a different way to the crisis than the networks of weakly connected companies. Besides the type of the network and its features, the optimal size of the time window to calculate cross-correlation has been investigated. The optimal window size is a month, T=20d. In the analysis based on the T=20d cross-correlation time window, the fluctuations are suppressed such that important trends can be seen and discussed. On the other hand, in the analysis performed for the shortest time window (*T* = 5 days), the averaged clustering coefficient and the transitivity for the network of the significantly connected companies decreases in the crises, while for the networks of strongly and typically connected companies, these parameters visibly increase. This situation might be the effect of the short window time wherein the Pearson correlation coefficient is calculated on the five data point sets. This observation supports the conclusion that the optimal time window for the analysis of the daily time series returns is a month period.

The presented results show that the proposed network structures are capable of describing and measuring the changes resulting from crises on the stock markets. Moreover, the introduced parameters, the rank network entropy and the cycle entropy, are useful parameters in the analysis of structure changes and crises recognition. Particularly, the rank entropy, which is capable of quantitatively characterising network structure changes and those parameters, might be useful in crash analysis. On the other hand, the introduced network structures, which are composed of strongly, significantly, typically and weakly correlated companies, do not introduce as strong of constraints as the frequently used MST structures. For example, the GE company, which is the centre of the MST in [16], is one of the highly connected companies here, but is not so prominent as in the MST structure. The number of links of GE is comparable to the median level of the number of links for a given network type.

Besides the main results of the paper, it has been observed that the rank entropy is likely to change its value in a step-like function, showing that, according to the market situation, the network will change to some well-established structures. This is a very intriguing hypothesis which deserves further study.

## Figures and Tables

**Figure A1:**
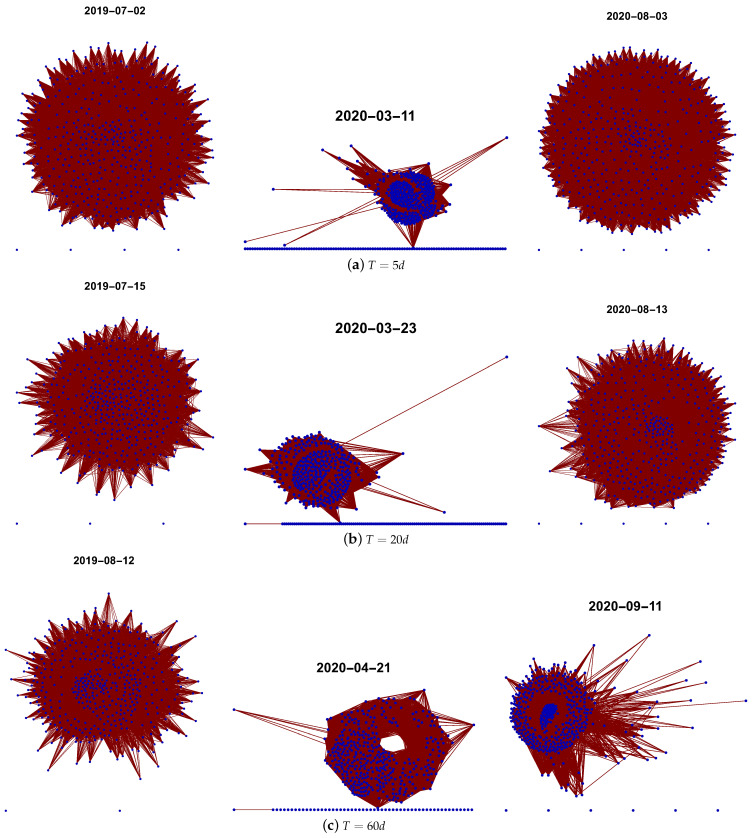
Examples of the graphs obtained for the network of strongly correlated companies. The presented graphs show the network state before and in the first and second stage of the COVID-19 pandemic. The top graphs correspond to the shortest time window T=5d, the middle three graphs represent networks for the month time window and the bottom graphs present the examples for the quarter time window.

**Figure A2:**
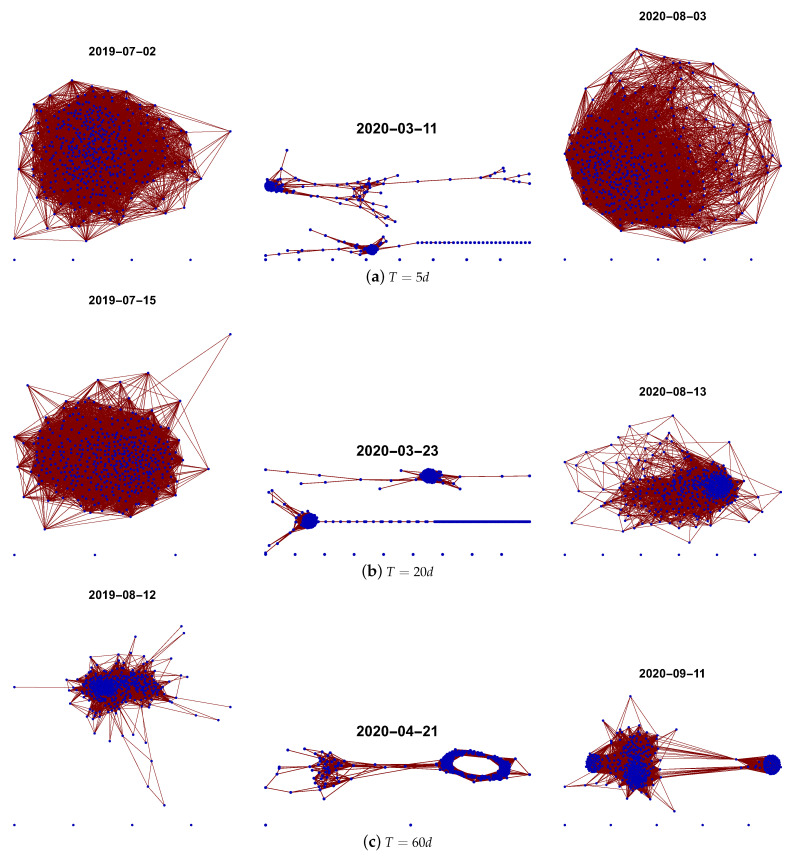
Examples of the graphs obtained for the network of weakly connected companies. The presented graphs show the network before and in the first and second stage of the COVID-19 pandemic. The top graphs correspond to the shortest time window T=5d, the middle three graphs represent networks for the month time window and the bottom graphs present the examples for the quarter time window.

**Figure A3:**
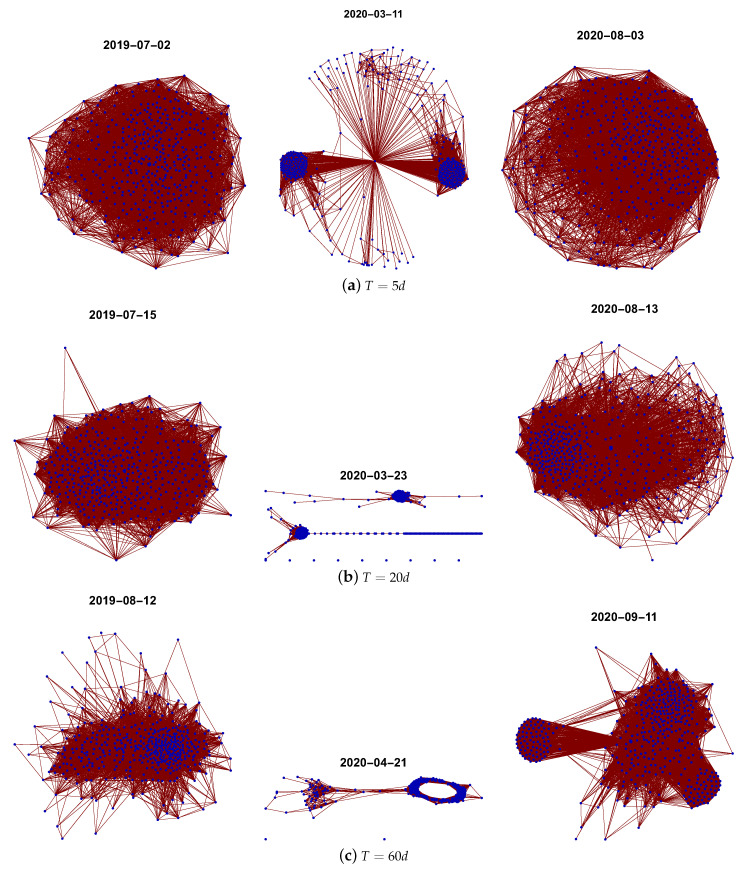
Examples of the graphs obtained for the network of the typically connected companies. The presented graphs show the network before and in the first and second stage of the COVID-19 pandemic. The top graphs correspond to the shortest time window T=5d, the middle three graphs represent networks for the month time window and the bottom graphs present the examples for the quarter time window.

**Figure A4:**
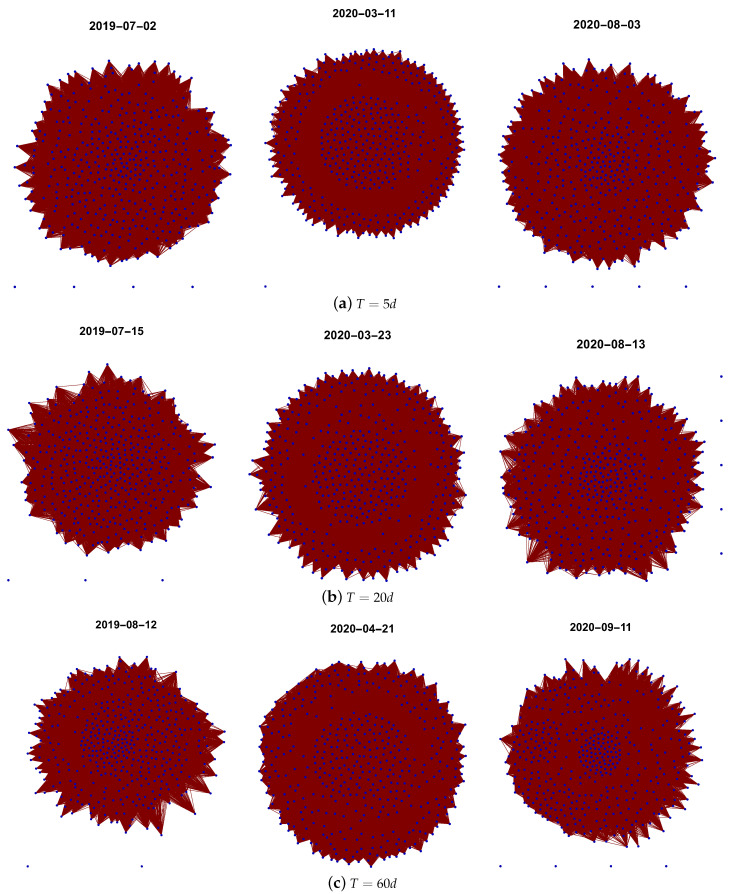
Examples of the graphs obtained for the network of significantly connected companies. The presented graphs show the network before and in the first and second stage of the COVID-19 pandemic. The top graphs correspond to the shortest time window T=5d, the middle three graphs represent networks for the month time window and the bottom graphs present the examples for the quarter time window.

## Data Availability

The data used in this study were downloaded from the web page www.stoog.pl (accessed on 28 March 2021).

## Appendix A. Companies List

The analysis of this paper is based on the following companies quotes (using the standard abbreviations):

A, AAL, AAP, AAPL, ABBV, ABC, ABT, ACN, ADBE, ADI, ADM, ADP, ADS, ADSK, AEE, AEP, AES, AFL, AIG, AIV, AIZ, AJG, AKAM, ALB, ALGN, ALK, ALL, ALLE, ALXN, AMAT, AMD, AME, AMG, AMGN, AMP, AMT, AMZN, ANSS, ANTM, AON, AOS, APA, APD, APH, APTV, ARE, ARNC, ATVI, AVB, AVGO, AVY, AWK, AXP, AYI, AZO, BA, BAC, BAX, BBY, BDX, BEN, BFb, BHF, BIIB, BK, BKNG, BKR, BLK, BLL, BMY, BPYU, BRKb, BSX, BWA, BXP, C, CAG, CAH, CAT, CB, CBOE, CBRE, CCI, CCL, CDNS, CERN, CF, CFG, CHD, CHRW, CHTR, CI, CINF, CL, CLX, CMA, CMCSA, CME, CMG, CMI, CMS, CNC, CNP, COF, COG, COO, COP, COST, COTY, CPB, CPRI, CRM, CSCO, CSX, CTAS, CTSH, CTXS, CVS, CVX, D, DAL, DD, DE, DFS, DG, DGX, DHI, DHR, DIS, DISCA, DISCK, DISH, DLR, DLTR, DOV, DRE, DRI, DTE, DUK, DVA, DVN, DXC, EA, EBAY, ECL, ED, EFX, EIX, EL, EMN, EMR, EOG, EQIX, EQR, EQT, ES, ESS, ETN, ETR, EW, EXC, EXPD, EXPE, EXR, F, FAST, FB, FBHS, FCX, FDX, FE, FFIV, FIS, FISV, FITB, FL, FLIR, FLR, FLS, FMC, FRT, FTI, FTV, GD, GE, GILD, GIS, GL, GLW, GM, GOOG, GOOGL, GPC, GPN, GPS, GRMN, GS, GT, GWW, HAL, HAS, HBAN, HBI, HCA, HD, HES, HIG, HLT, HOG, HOLX, HON, HP, HPE, HPQ, HRB, HRL, HSIC, HST, HSY, HUM, IBM, ICE, IDXX, IFF, ILMN, INCY, INFO, INTC, INTU, IP, IPG, IPGP, IR, IRM, ISRG, IT, ITW, IVZ, J, JBHT, JCI, JEF, JNJ, JNPR, JPM, JWN, K, KDP, KEY, KHC, KIM, KKR, KLAC, KMB, KMI, KMX, KO, KR, KSS, KSU, L, LB, LEG, LEN, LH, LHX, LKQ, LLY, LMT, LNC, LNT, LOW, LRCX, LUMN, LUV, LYB, M, MA, MAA, MAC, MAR, MAS, MAT, MCD, MCK, MCO, MDLZ, MDT, MET, MGM, MHK, MKC, MMC, MMM, MNST, MO, MOS, MPC, MRK, MRO, MS, MSFT, MSI, MTB, MTD, MU, NAVI, NCLH, NDAQ, NEE, NEM, NFLX, NI, NKE, NLOK, NLSN, NOC, NOV, NRG, NSC, NTAP, NTRS, NVDA, NWL, NWS, NWSA, O, OKE, OMC, ORCL, ORLY, OXY, PAYX, PBCT, PCAR, PCG, PDCO, PEAK, PEG, PEP, PFE, PFG, PG, PGR, PH, PHM, PKG, PKI, PLD, PM, PNC, PNR, PNW, PPG, PPL, PRGO, PRU, PSA, PSX, PVH, PWR, PXD, PYPL, QCOM, QRVO, RCL, RE, REG, REGN, RF, RHI, RL, RMD, ROK, ROP, ROST, RRC, RSG, RTX, SBAC, SBUX, SCHW, SEE, SHW, SIG, SJM, SLB, SLG, SNA, SNPS, SO, SPG, SPGI, SRCL, SRE, STT, STX, STZ, SWK, SWKS, SYF, SYK, SYY, T, TAP, TDG, TEL, TFC, TGT, TJX, TMO, TNL, TRIP, TRV, TSCO, TSN, TXN, TXT, UA, UAA, UAL, UDR, UHS, ULTA, UNH, UNM, UNP, UPS, URI, USB, V, VAR, VFC, VIAC, VLO, VMC, VNO, VRSK, VRSN, VRTX, VTR, VTRS, VZ, WAT, WBA, WDC, WEC, WELL, WFC, WHR, WLTW, WM, WMB, WMT, WRK, WU, WY, WYNN, XEC, XEL, XLNX, XOM, XRAY, XRX, XYL, YUM, ZBH, ZION, ZTS.

## Appendix B. Graph Examples

Here, a few of the network examples generated and analysed in the study are presented (Figure A1, Figure A2, Figure A3 and Figure A4). The figures were obtained using Mathematica 11 with the “SpringElectricalEmbeding” algorithm. This algorithm optimises the position of nodes with respect to its rank. However, due to the number of nodes and links, the graphs are a bit unclear, particularly in the case of the network of significantly connected companies in Figure A4. However, even a cursory observation shows that the proposed structures give significantly different results. Particularly, networks representing the state of the stock market during the pandemic vary significantly, even though loss of network connectivity is observed. It goes beyond the scope of this paper, but the detailed analysis of the network’s evolution from the point of view of the role of a particular company or the reaction of a group of companies to the pandemic situation might be very interesting; however, this is left for another study.
